# Effects of short-term exercise and endurance training on skeletal muscle mitochondria damage induced by particular matter, atmospherically relevant artificial PM2.5

**DOI:** 10.3389/fpubh.2024.1302175

**Published:** 2024-02-28

**Authors:** Wenduo Liu, Zilin Wang, Yu Gu, Han-Sol So, Sung-Ho Kook, Yoonjung Park, Sang Hyun Kim

**Affiliations:** ^1^Department of Sports Science, College of Natural Science, Jeonbuk National University, Jeonju, Republic of Korea; ^2^Department of Bioactive Material Sciences, Research Center of Bioactive Materials, Jeonbuk National University, Jeonju, Republic of Korea; ^3^Laboratory of Integrated Physiology, Department of Health and Human Performance, University of Houston, Houston, TX, United States

**Keywords:** endurance training, particulate matter, aerobic fitness, mitochondria, fine dust

## Abstract

**Introduction:**

This study aimed to investigate the potential of short-term aerobic exercise to mitigate skeletal muscle mitochondrial damage following ambient PM2.5 exposure, and how 12 weeks of endurance training can enhance aerobic fitness to protect against such damage.

**Methods:**

Twenty-four male C57BL/6 J mice were split into sedentary (SED, *n* = 12) and endurance training (ETR, *n* = 12) groups. The ETR group underwent 12 weeks of training (10–15 m/min, 60 min/day, 4 times/week), confirmed by an Endurance Exercise Capacity (EEC) test. Post-initial training, the SED group was further divided into SSED (SED and sedentary, *n* = 6) and SPE (SED and PM2.5 + Exercise, *n* = 6). Similarly, the ETR group was divided into EEX (ETR and Exercise, *n* = 6) and EPE (ETR and PM2.5 + Exercise, *n* = 6). These groups underwent 1 week of atmospherically relevant artificial PM2.5 exposure and treadmill running (3 times/week). Following treatments, an EEC test was conducted, and mice were sacrificed for blood and skeletal muscle extraction. Blood samples were analyzed for oxidative stress indicators, while skeletal muscles were assessed for mitochondrial oxidative metabolism, antioxidant capacity, and mitochondrial damage using western blot and transmission electron microscopy (TEM).

**Results:**

After 12 weeks of endurance training, the EEC significantly increased (*p* < 0.000) in the ETR group compared to the SED group. Following a one-week comparison among the four groups with atmospherically relevant artificial PM2.5 exposure and exercise treatment post-endurance training, the EEX group showed improvements in EEC, oxidative metabolism, mitochondrial dynamics, and antioxidant functions. Conversely, these factors decreased in the EPE group compared to the EEX. Additionally, within the SPE group, exercise effects were evident in HK2, LDH, SOD2, and GPX4, while no impact of short-term exercise was observed in all other factors. TEM images revealed no evidence of mitochondrial damage in both the SED and EEX groups, while the majority of mitochondria were damaged in the SPE group. The EPE group also exhibited damaged mitochondria, although significantly less than the SPE group.

**Conclusion:**

Atmospherically relevant artificial PM2.5 exposure can elevate oxidative stress, potentially disrupting the benefits of short-term endurance exercise and leading to mitochondrial damage. Nonetheless, increased aerobic fitness through endurance training can mitigate PM2.5-induced mitochondrial damage.

## 1 Introduction

Physical fitness and air pollution are two significant factors that affect human health (1, 2). Particulate matter (PM), which consists of minuscule particles capable of deeply penetrating the lungs upon inhalation, is a form of air pollution linked to various health issues (3, 4). PM is generally classified into PM10 (10 μm or less), PM2.5–10 (2.5–10 μm) and PM2.5 (2.5 μm or less) based on differences in diameter (5). The smaller the particle size, the deeper it can penetrate, which can adversely affect not only the lungs but also the heart and other tissues (6). Accordingly, the effects of PM2.5 on health are currently being studied in various tissues, including lung (7, 8), heart (7, 9), adipose tissue (10), brain (11), and skeletal muscle (12, 13). Most of these studies suggest that PM2.5 induced oxidative stress contributes to a variety of health problems, including cardiovascular function and insulin resistance (7, 14–17). In addition, most existing studies have been conducted through epidemiological or cross-sectional investigations (6, 17–19). However, experimental studies based on exposure to PM2.5 have also relied on methods such as instillation via the nose or trachea and intravenous or intraperitoneal injections, rather than whole-body inhalation exposure. Recently, studies on real ambient PM2.5 inhalation have been reported, but most of them are related to the cardiovascular system (20–22), adipose tissue (10, 23), brain (11) etc., and studies focusing on skeletal muscle are very scarce.

Skeletal muscle is the largest tissue in our body and is not only the subject of movement, but also a plastic tissue that can rapidly modify its structure, function, and metabolism in response to various stresses applied internally or externally (24, 25). Therefore, skeletal muscle health is the most fundamental for improving human metabolism, physical activity ability, and quality of life (24). Skeletal muscle function and metabolism are essential for aerobic fitness, enabling individuals to engage in various activities, exercise, and maintain overall health and vitality (26). The interdependence between metabolism, and mitochondrial function in skeletal muscle underscores the pivotal role of mitochondria in sustaining proper skeletal muscle function (27–29). Mitochondria, specialized organelles responsible for ATP production, directly influence the efficiency and effectiveness of skeletal muscle activity (30, 31). Disruption or alteration of mitochondrial function can directly impact metabolic processes and overall skeletal muscle function (32). Exercise is necessary to prevent this, especially aerobic exercises such as running, which is effective (33). Acute aerobic exercise also increases metabolism in skeletal muscle, leading to an increased expression of skeletal muscle mitochondrial electron transport enzymes and enhanced glucose transporter protein type-4 (GLUT4) levels (34–37). It is a well-known fact that long-term regular endurance training offers numerous benefits for preventing or treating various health problems (38, 39). However, when exercising during PM2.5 exposure or following inhalation, inflammatory chemicals and toxic substances contained in fine dust can easily penetrate the peripheral tissues (9, 21). This is because the smaller the particle size of PM, the deeper it can penetrate the human body (5). Our preliminary data also indicate that skeletal muscle mitochondria in mice exposed to PM2.5 are damaged (Supplementary Figure 1). This led us to ask the following questions: Does exercise after PM2.5 inhalation exacerbate or prevent the adverse health effects of PM2.5? Can aerobic fitness, improved by long-term regular endurance training, prevent adverse health effects caused by PM2.5 inhalation?

The purpose of this study was to determine whether short-term aerobic exercise can diminish PM2.5 inhalation-induced skeletal muscle mitochondria damage, and how enhanced aerobic fitness by 12 weeks of endurance training protects mitochondrial damage against PM2.5 exposure. The study was conducted in an environment where mice were exposed to atmospherically relevant artificial PM2.5, with a specific focus on examining the role of skeletal muscle mitochondria.

## 2 Methods

### 2.1 Experimental procedures

In this study, 4-week-old male C57BL/6 J mice were purchased and tested after undergoing a 1-week acclimation period. The room was maintained at a controlled temperature (18–22°C) and humidity (40–60%) on a 12-h light–dark cycle. Water and feed were provided *ad libitum* during the breeding process. At 5 weeks of age, the mice were randomly assigned to two groups: sedentary (SED, *n* = 12) and endurance training (ETR, *n* = 12). The mice were then treated for a duration of 12 weeks. Body weight measurements were taken weekly throughout the training period. At the end of the 12-week training period, the SED group was further categorized into the SSED (SED and sedentary, *n* = 6) and SPE (SED and Exercise after PM2.5 exposure, *n* = 6) groups, while the ETR group was categorized into the EEX (ETR and Exercise, *n* = 6) and EPE (ETR and Exercise after PM2.5 exposure, *n* = 6) groups. Following the 12-week training period, each group was further categorized into four subgroups, which performed either exercise or ambient PM2.5 exposure combined with exercise for 1 week. The exercise and atmospherically relevant artificial PM2.5 exposure were conducted three times, with a total of three sessions, on alternate days starting from the second day after the completion of the 12-week exercise period. Exercise was performed immediately after PM2.5 exposure, and the exercise protocol was set identically to the last exercise of 12 weeks of endurance training. After the completion of all treatments, skeletal muscle was harvested at 18 h after the last exercise session. An overview of the experimental design can be found in the Supplementary Figure 2. All experimental procedures were conducted in accordance with the guidelines and regulations set by the Institutional Animal Care and Use Committee of Jeonbuk National University (IACUC approval no. CBNU-2022-0067).

### 2.2 Atmospheric simulation chamber (ASC) system

The ASC system is a whole-body exposure device designed to replicate the inhalation of PM2.5 in the atmosphere while maintaining a consistent average concentration. The PM solution was formulated by mixing 10 organic and inorganic compounds, including oxalic acid, malonic acid, glutaric acid, sucrose, 2,5-dihydroxybenzoic acid, glycine, ammonium sulfate, ammonium nitrate, acetate, and glycerol, into distilled water. The PM is aerosolized using a nebulizer (TQ-50-C0,5; Meinhard, USA) and, subsequent to passing through a polypropylene melt-blown filter, particles larger than 2.5 μm are sieved out and entered the chamber. The concentration of PM2.5 within the chamber is continuously monitored in real-time using a particle counter (BT-610; Met One, USA), with the predetermined concentration automatically upheld through a flow controller program. SPE and EPE group were exposed to PM2.5 for 2 h per day, three times a week, at a concentration of 50.1 ± 8.1 μg/m^3^. The chamber maintained a humidity level of 55–60% and a temperature range of 23–25°C.

### 2.3 Chemical composition

The organic components and inorganic salts for the preparation of artificial PM2.5 are listed in Supplementary Table 1. Eight organic compounds with carboxylic acid, polyol, sugar, aromatic, and amino acid functional groups were investigated. Ammonium sulfate and ammonium nitrate were used as the model inorganic salts because of their abundance in air (40–43). The 10 compounds in Supplementary Table 2 were mixed at an organic to inorganic dry mass ratio of 1:1 to mimic the chemical complexity of atmospheric aerosols. The compounds were purchased from Sigma-Aldrich (purity ≥98%) and were used without further purification. The mixture of 10 components was dissolved in purified water.

### 2.4 Endurance training protocol

Endurance training was conducted using a treadmill. Prior to the initiation of the 12-week program, all mice underwent treadmill adaptation exercise for 1 week, which consisted of running at a speed of 10 m/min for 20–30 min, three times per week.

Following the adaptation treadmill exercise, the exercise group received a training program on the treadmill with a 0% incline. One-time exercise program included a 10-min warm-up period at a speed of 10 m/min, a 45-min main exercise period at a speed of 15 m/min, and a 5-min cool-down period at a speed of 10 m/min. This program was repeated 4 times per week. The speed for the main exercise period was set at 60–70% of the maximum running speed determined through the endurance exercise capacity (EEC) test, as described (44). EEC test for setting running speed was conducted after the mice had adapted to the treadmill.

### 2.5 Endurance exercise capacity (EEC) test

The progressive exercise test for EEC was conducted on all mice 24 h after the completion of the 12-week exercise program to assess the impact of endurance training. The EEC test was performed on a treadmill with a fixed slope of 15 degrees. Initially, the treadmill speed was set at 10 m/min for the first 5 min, and subsequently, the speed was increased by 2 m/min every minute. During the test, if a mouse was unable to continue running despite sponge stimulation and remained on the electric shock grid for more than 3 s, the test was stopped. The total running time, vertical distance, and work were measured or calculated using methodologies described in previous studies (45).

### 2.6 Thiobarbituric acid-reactive substances (TBARS)

TBARS, an oxidative stress indicator, was analyzed after storing the isolated gastrocnemius muscle at −80°C until analysis. Freeze-clamped muscle samples were homogenized in ice-cold buffer (50 mM sodium phosphate, pH 7.0) and centrifuged (13,000 g, 10 min, 4°C) to separate the supernatant. The supernatant was analyzed according to the manual of the Quantichrom TBARS Assay Kit (Bioassay Systems, Hayward, CA, USA) (35).

### 2.7 Westernblot analysis

The analysis of protein expression was conducted using the gastrocnemius muscle (GAS), which was rapidly frozen in liquid nitrogen upon extraction and subsequently stored at −80°Celsius. The GAS samples were homogenized in a cold buffer [50 mM Tris·HCl (pH 7.4), 1% NP-40, 0.25% sodium deoxycholate, 150 mM NaCl, 1 mM ethylenediaminetetraacetic acid (EDTA, pH 7.4), 1 mM Pefabloc (Roche, Basel, Switzerland), 1 mM NaF, 1 μg/mL aprotinin, 1 μg/mL leupeptin, 1 μg/mL pepstatin, 0.1 mM bpV(phen), and 2 mg/mL β-glycerophosphate] kept on ice.

The homogenate was solubilized in Laemmli sample buffer after determining the protein concentration using the Bradford (BIO-RAD, Bio-Rad Protein Assay Dye Reagent Concentrate, CA, USA) (46). Following gel electrophoresis, each sample was transferred onto nitrocellulose membranes and blocked with skim milk at room temperature. Subsequently, the membranes were incubated overnight at 4°C with primary antibodies [Peroxisome proliferator-activated receptor gamma coactivator 1-alpha (PGC-1α, GTX37356, GeneTex, CA, USA); NADH:ubiquinone oxidoreductase subunit A9 (NADH, ab110242, Abcam, MA, USA); succinate dehydrogenase B (SDHB, ab14714, Abcam); cytochrome oxidase (COX) subunit I (COX I, ab14705, Abcam,); GLUT4 (sc-53566, Santa Cruz Biotecnology, CA, USA); Hexokinase 2 (HK2, Santa Cruz Biotecnology, sc-374091); phosphofrucokinase (PFK, sc-166722, Santa Cruz Biotecnology), lactate dehydrogenase (LDH, sc-133123, Santa Cruz Biotecnology), mitofusin 1 (MFN1, sc-166644, Santa Cruz Biotecnology), MFN2 (sc-515647, Santa Cruz Biotecnology), optic atrophy 1 (OPA1, sc-393296, Santa Cruz Biotecnology), mitochondria fission 1 protein (Fis1, sc-376447, Santa Cruz Biotecnology), dynamin-related protein 1 (Drp1, sc-32898, Santa Cruz Biotecnology), superoxide dismutase (SOD) type 1 (SOD1, sc-8637, Santa Cruz Biotechnology), SOD2 (sc-18503, Santa Cruz Biotechnology); catalase (sc-271358, Santa Cruz Biotechnology); glutathione peroxidase 4 (GPX4, Santa Cruz Biotechnology), Bcl2-associated X protein (BAX, sc-7480, Santa Cruz Biotechnology), B-cell lymphoma 2 (Bcl2, sc-7382, Santa Cruz Biotechnology), Parkin (sc-32282, Santa Cruz Biotecnology), PTEN-induced kinase 1 (PINK1, sc-517353, Santa Cruz Biotecnology), β-actin (MA1-140 Invitrogen, MN, USA)], and then subjected to further incubation with appropriate secondary antibodies [mouse anti-goat (sc-2354, Santa Cruz Biotechnology); mouse anti-rabbit (sc-2357, Santa Cruz Biotechnology); goat anti-mouse (sc-2005, Santa Cruz Biotechnology)] for protein detection. Protein visualization was conducted using the ECL Western Blotting Detection Reagent (GE Healthcare, Chalfont St Giles, UK), and quantification was carried out using the ChemiDoc XRS+ system (BIO-RAD, Hercules, CA, USA).

### 2.8 Transmission electron microscopy

Transmission electron microscopy (TEM) was utilized to examine the mitochondrial structure. The soleus muscle was fixed with a solution containing 2.5% glutaraldehyde and 4% formaldehyde in 0.1 M phosphate buffer at pH 7.4 for a duration of 2 h immediately after extraction. Subsequently, the fixed muscles were post-fixed with 1% osmium tetroxide for 2 h. The muscles were then dehydrated using a graded series of ethanol and embedded in Epon-812 resin. Sections were obtained using a NOVA ultramicrotome (LKB, Vienna, Austria) and mounted on a 100-mesh grid. Thin sections with a thickness of approximately 80 nm were prepared for TEM. The sections were stained with 0.1% toluidine blue for light microscopy. For the electron microscope, the sections were stained with 0.1% toluidine blue. Thin sections with a thickness of approximately 80 nm for TEM were cut from both transverse and longitudinal planes using a NOVA ultramicrotome (LKB, Vienna, Austria) and placed on a 100-mesh grid. To enhance visualization, the sections were stained with uranyl acetate and lead citrate and examined using an electron microscope (H7650, accelerating voltage: 80 kV, Hitachi, Japan) to confirm the specimens. TEM samples were analyzed by Transmission Electron Microscope (JEM-2010, JEOL) installed in the Center for University-Wide Research Facilities (CURF) at Jeonbuk National University.

### 2.9 Statistics analysis

All data were presented as mean ± standard deviation (SD). Normality and homogeneity of variances were assessed using Shapiro–Wilk’s and Levene’s tests, respectively. Two-way repeated ANOVA was conducted to analyze the body weight change between the SED and ETR groups over the 12-week endurance training. In order to verify the effects of endurance training for 12 weeks, an independent t-test was performed to compare the SED and ETR groups. Comparisons between groups (SSED, SPE, EEX, and EPE) after a week of PM2.5 and exercise concurrent treatment following endurance training were performed with one-way ANOVA followed by Tukey’s *post hoc* test to confirm a significant difference (*p* < 0.05).

## 3 Results

### 3.1 PM2.5 exposure and exercise on endurance exercise capacity (EEC)

**Figure 1:**
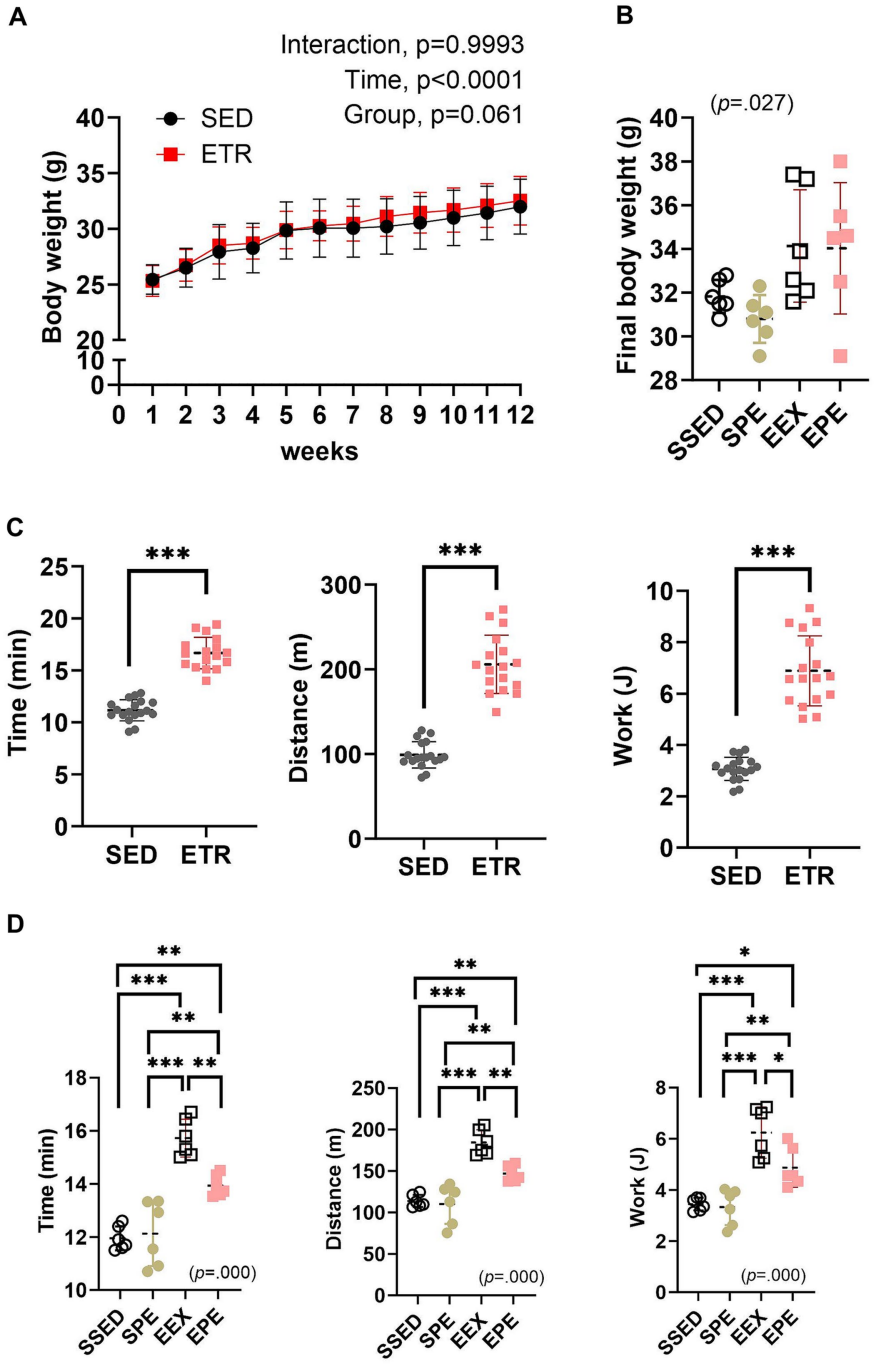
Adminstration of ambient PM2.5 exposure and aerobic exercise decreases the enhanced endurance exercise capacity (EEC) observed after 12 weeks of endurance training. **(A)** Body weight changes after 12 weeks of endurance training were compared between the groups by the two-way repeated measures ANOVA (*n* = 12). **(B)** Final body weight following 1 week of PM2.5 inhalation and aerobic exercise in conjunction with 12 weeks of endurance training were compared between the groups by the one-way ANOVA (*n* = 6). **(C)** EEC 12 weeks of endurance training were compared between the groups by independent samples *t*-test (*n* = 12). ***p < 0.001 between the groups. **(D)** EEC following 1 week of PM2.5 inhalation and aerobic exercise in conjunction with 12 weeks of endurance training were compared between the groups by the one-way ANOVA (*n* = 6). *p < 0.05, **p < 0.01, ***p < 0.001 between the groups. SED, sedentary group; ETR, endurance training group; SSED, SED and sedentary group; SPE, SED and exercise after PM exposure; EEX, ETR and exercise; EPE, ETR and exercise after PM2.5. All data are presented as Mean ± SD.

There was no significant change in body weight during the 12 weeks of endurance training (Figure 1A). However, a significant difference (*p* = 0.027) in the final body weight between groups was observed following 1 week of ambient PM2.5 exposure and treadmill running after the 12 weeks of endurance training. Nonetheless, post-hoc analysis did not reveal any significant differences, but the EEX (*p* = 0.053) and EPE (*p* = 0.063) groups showed a higher trend compared to the SPE group (Figure 1B).

The significantly improved (*p* < 0.000) EEC following endurance training is presented in Figure 1C. These results indicate the effectiveness of the 12 weeks of endurance training conducted in this study. However, the increased EEC was attenuated by 1 week of PM2.5 exposure and exercise (EPE group) (Figure 1D). In contrast, in the SPE group, there were no significant changes compared to the SSED (Figure 1D).

### 3.2 PM2.5 exposure and exercise on oxidative metabolism in skeletal muscle

**Figure 2:**
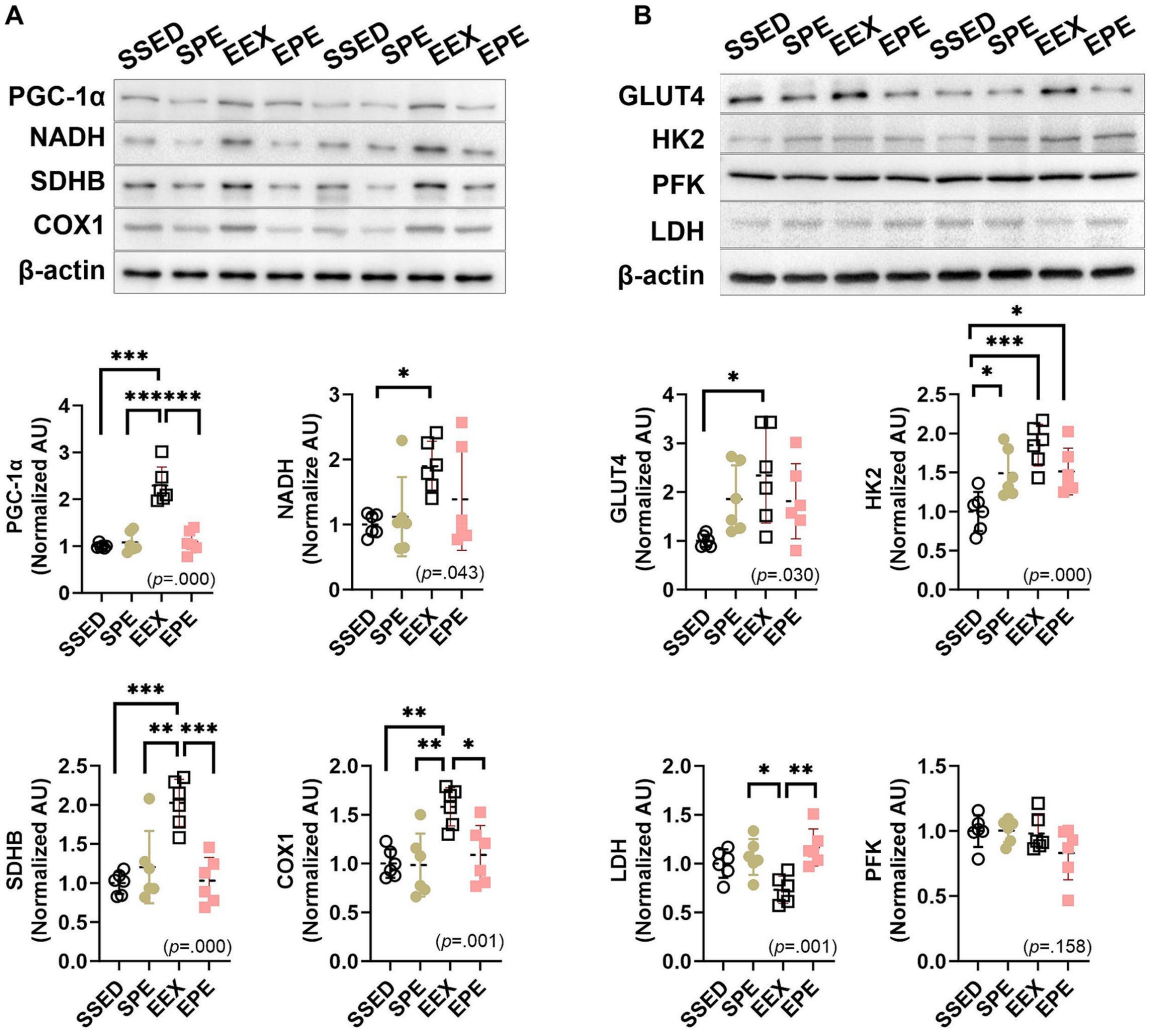
Administration of PM2.5 and aerobic exercise decreases the enhanced skeletal muscle oxidative metabolism observed after endurance training. **(A)** The expression of PGC-1α and mitochondrial enzymes was compared between the groups following 1 week of PM_2.5_ inhalation and treadmill exercise in conjunction with 12 weeks of endurance training (*n* = 6). **(B)** The expression of glucose metabolism-related proteins was compared between the groups following 1 week of PM2.5 inhalation and treadmill exercise in conjunction with 12 weeks of endurance training (*n* = 6). SSED, SED and sedentary group; SPE, SED and exercise after PM exposure; EEX, ETR and exercise; EPE, ETR and exercise after PM2.5. All data are presented as Mean ± SD. *p < 0.05, **p < 0.01, ***p < 0.001 between the groups.

As shown in Figure 1, similar to the increased EEC, skeletal muscle oxidative metabolism was also increased by endurance training (Figure 2).

PGC-1α, which ultimately induces mitochondrial respiration and fatty acid oxidation by upregulating the expression of oxidative phosphorylation (OXPHOS) and fatty acid β-oxidation genes, as well as mitochondrial respiratory enzymes NADH, SDHB, and COX1, demonstrated an increase in the EEX group compared to SSED (*p* < 0.001) (Figure 2A). These endurance training effects were influenced by an administration of ambient PM2.5 exposure and exercise (SPE or EPE group) for an additional 1 week. In the EPE group, PGC-1α, SDHB, and COX1 decreased significantly compared to the EEX group (*p* < 0.001), and although NADH did not show a significant difference, it demonstrated a decreasing trend. Moreover, the group that underwent PM2.5 and exercise for 1 week without endurance training, the SPE group, did not exhibit any discernible exercise effect and showed a similar level to the EPE group (Figure 2A).

The results related to glucose metabolism following endurance training and concurrent PM2.5 and exercise treatment are presented in Figure 2B. Endurance training resulted in an increase in GLUT4 and HK2 (*p* < 0.05) and a decrease in LDH (*p* < 0.05). However, there was no significant difference in the glycolysis enzyme, PFK. These changes induced by endurance training were somewhat higher in the groups with PM2.5 exposure and exercise administration, the SPE and EPE groups, compared to the SSED group for GLUT4, but the differences were not significant. However, for HK2, both the SPE and EPE groups showed a significant increase compared to SSED (*p* < 0.05), although they decreased (not significant) compared to the EEX group. Similarly, LDH showed a significant increase (*p* < 0.01) in both the SPE and EPE groups compared to EEX and showed a level similar to SSED.

### 3.3 PM2.5 exposure and exercise on mitochondrial dynamics and mitochondrial damage

**Figure 3:**
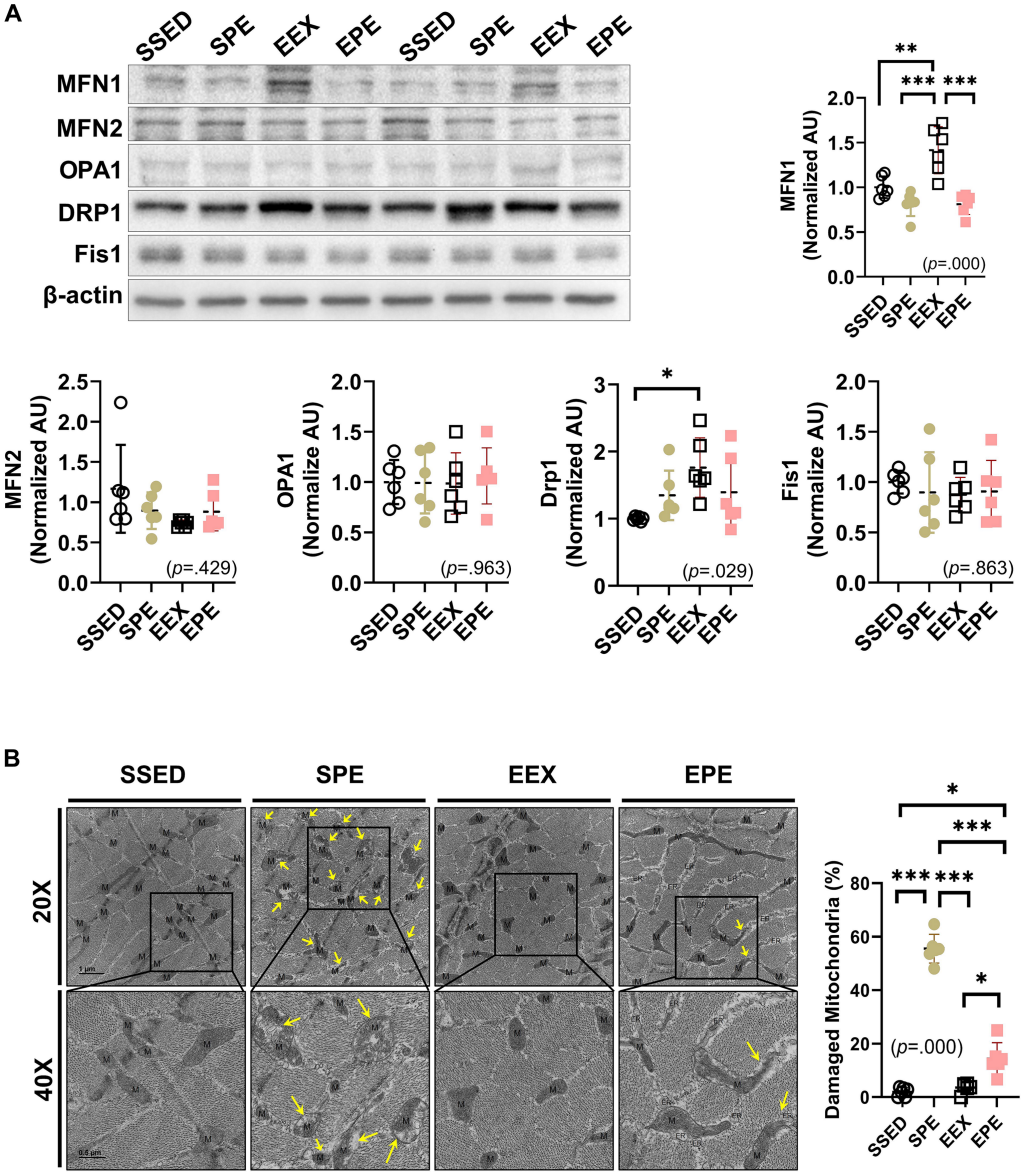
Changes in skeletal muscle mitochondrial dynamics and mitophagy-related proteins were assessed following concurrent treatment of PM2.5 and treadmill exercise for 1 week after 12 weeks of endurance training. **(A)** The expression of mitochondrial dynamics was compared between the groups following 1 week of PM2.5 inhalation and treadmill exercise in conjunction with 12 weeks of endurance training, using one-way ANOVA with Tukey’s *post hoc* test (*n* = 6). **(B)** Representative transmission electron microscopy (TEM) images and the ratio of damaged mitochondria to total mitochondria in skeletal muscle were examined following 1 week of ambient PM2.5 exposure and treadmill exercise, in conjunction with 12 weeks of endurance training. One representative mitochondrion is indicated by M, and damaged mitochondria, such as swollen cristae or loss of cristae are indicated by yellow arrows. All data are presented as Mean ± SD. *p < 0.05, **p < 0.01, ***p < 0.001 between the groups. SSED, SED and sedentary group; SPE, SED and exercise after PM exposure; EEX, ETR and exercise; EPE, ETR and exercise after PM2.5 exposure.

The results related to mitochondrial dynamics following concurrent PM2.5 and exercise treatment after endurance training are depicted in Figure 3A. In the EEX group, the mitochondrial fusion-promoting factor MFN1 (*p* < 0.01) and the mitochondrial fission-promoting factor DRP1 (*p* < 0.05) increased compared to the SSED group. However, in the EPE group, which received administration of ambient PM2.5 exposure and exercise after endurance training, these factors were decreased compared to the EEX group, and were at a level similar to the SSED group. Another fusion/fission-promoting factor, MFN2, OPA1, and Fis1, showed no significant differences among groups.

The presence or absence of mitochondrial damage was verified by TEM images. Damaged mitochondria, such as swollen cristae or loss of cristae, were indicated by yellow arrows, particularly prominent in the SPE group (Figure 3B, TEM images). The calculated ratio of damaged mitochondria to total mitochondria also revealed a significantly higher proportion in the SPE group compared to SSED, EEX, and EPE (*p* < 0.000). In contrast, the ratio of damaged mitochondria in EPE was much higher (*p* < 0.05) than in SSED and EEX but noticeably lower (*p* < 0.000) than in SPE. However, in the EPE group subjected to the same ambient PM2.5 exposure and exercise in the same way as the SPE group, there was no significant morphological difference from the SSED and EEX groups (Figure 3B right dot graph).

### 3.4 PM2.5 exposure and exercise on oxidative stress and mitophagy

**Figure 4:**
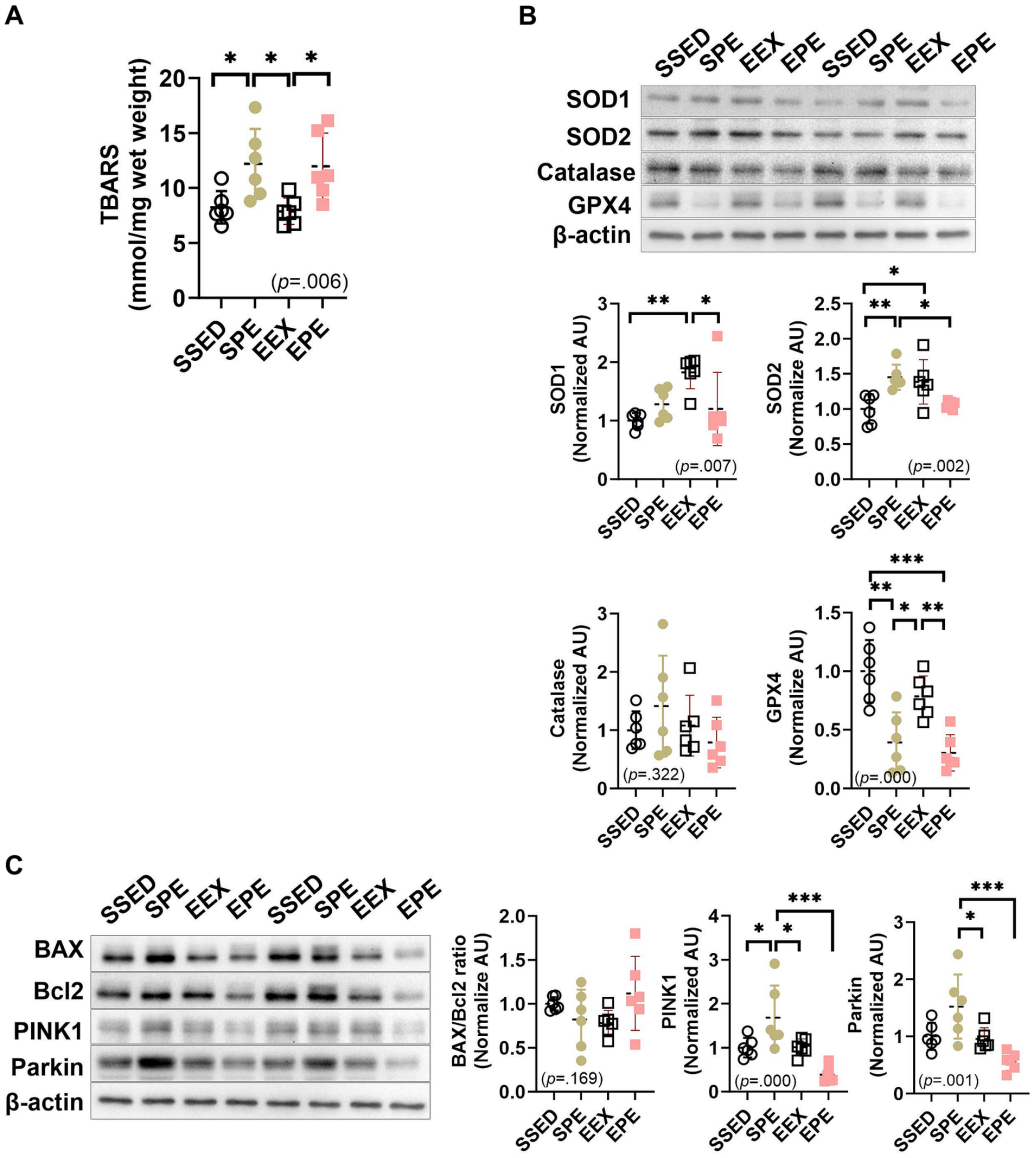
Administration of ambient PM2.5 exposure and treadmill exercise for 1 week, after 12 weeks of endurance training, increased skeletal muscle oxidative stress and induced mitophagy. **(A)** Skeletal muscle TBARS level, an indicator of oxidative stress. The expression of **(B)** antioxidant enzymes and **(C)** mitophagy-related proteins were compared between the groups following 1 week of ambient PM2.5 exposure and treadmill exercise in conjunction with 12 weeks of endurance training (*n* = 6). All data are presented as Mean ± SD. *p < 0.05, **p < 0.01, ***p < 0.001 between the groups. SSED, SED and sedentary group; SPE, SED and exercise after PM exposure; EEX, ETR and exercise; EPE, ETR and exercise after PM2.5 exposure.

Administration of atmospherically relevant artificial PM2.5 exposure and exercise after endurance training-induced changes in oxidative stress, antioxidants, and mitophagy in skeletal muscle, as shown in Figure 4.

Skeletal muscle TBARS levels, an indicator of oxidative stress, are shown in Figure 4A. There was no significant effect observed with long-term endurance (EEX group). SPE and EPE groups that administered short-term ambient PM2.5 exposure and exercise increased compared to SSED and/or EEX. In the case of the SPE group (*p* < 0.05), it was significantly higher than SSED and EEX, and the EPE group (*p* < 0.05) increased significantly compared to EEX.

In the case of antioxidant enzymes in Figure 4B, SOD1 (*p* < 0.01) and SOD2 (*p* < 0.05) were found to be increased in the EEX group compared to the SSED group. However, these increases were diminished in the EPE group, which received additional concurrent treatment of PM2.5 and exercise during the week following 12 weeks of endurance training. Specifically, in the EPE group, compared to the EEX group, SOD1 (*p* < 0.05) decreased significantly, while SOD2 (*p* = 0.065) showed a tendency to decrease. Furthermore, the decrease observed in the EPE group compared to the EEX group was also significant for GPX-4 (*p* < 0.01), and this reduction showed a significant difference (*p* < 0.001) even compared to the SSED group. On the other hand, in the group that underwent concurrently PM2.5 and exercise for 1 week without endurance training, known as the SPE group, SOD2 increased (*p* < 0.01) compared to the SSED group, while GPX4 decreased (*p* < 0.01). However, there was no significant difference in SOD1 and catalase.

Regarding apoptosis and mitophagy, as shown in Figure 4C, there was no significant difference in the BAX/Bcl-2 ratio between groups. PINK1 and Parkin, which promote mitophagy, were different depending on the presence or absence of endurance training when administration of ambient PM2.5 exposure and exercise. PINK1 was increased in the SPE group compared to the SSED (*p* < 0.05), EEX (*p* < 0.05), and EPE (*p* < 0.001) groups, and there was no difference between the SSED, EEX, and EPE groups. Parkin was higher than SSED in SPE group, but there was no significant difference, and there was a significant increase compared to EEX (*p* < 0.05) and EPE (*p* < 0.001).

## 4 Discussion

The present study aimed to determine whether short-term aerobic exercise after atmospherically relevant artificial PM2.5 exposure can prevent health risks caused by PM2.5 inhalation, and whether such effects are related to the improvement of aerobic fitness through endurance training before PM2.5 exposure and exercise administration. To achieve this, a 12-week endurance training using a treadmill was conducted in normal air to enhance aerobic fitness in mice, as shown in Figure 1C. Running time, vertical distance, and total work were significantly increased compared to sedentary mice (*p* < 0.001), indicating improved EEC. These results align with previous studies (47) using a similar exercise program and demonstrate the effectiveness of the current endurance training program.

To examine the benefits of enhanced aerobic fitness in response to short-term PM2.5 exposure and exercise administration, mice were exposed to atmospherically relevant artificial PM2.5 using the ASC system and performed treadmill exercise. As shown in Figure 1B, there was little change in body weight. In terms of EEC, the EPE group (PM2.5 exposure and exercise administration group after endurance training) showed a decrease compared to the EEX group that only underwent treadmill exercise. However, it still maintained a higher level than the sedentary group (SSED group). In contrast, the group that underwent ambient PM2.5 exposure and exercise administration without endurance training (SPE group) showed a similar level with SSED. However, unlike previous research (36, 37) showing improved skeletal muscle OXPHOS and running endurance after short-term aerobic exercise, such effects were not observed in the current study. Furthermore, both long-term endurance training and acute aerobic exercise sessions enhance oxidative metabolism, such as inducing an increase in OXPHOS via PGC-1α in skeletal muscle or promoting GLUT4 biogenesis (36, 37). Fatty acids and glucose enable OXPHOS (48), and the present study also confirmed increased OXPHOS in the EEX group (Figure 2). Lipid and glucose metabolism primarily rely on mitochondria to produce cellular energy (49). In the EEX group, PGC-1α, mitochondrial enzymes, GLUT4, and HK2 increased, while LDH, an enzyme that converts pyruvate to lactate in the final step of glycolysis (50), decreased (Figure 2). However, similar to the EEC results shown in Figure 1, skeletal muscle lipid and glucose metabolism were impaired in the EPE group compared to the EEX group, and the SPE group showed an increase in HK2 compared to the SSED group, but overall differences were insignificant. Thus, it means that aerobic exercise immediately after ambient PM2.4 exposure has no advantages, such as enhancement of EEC and promotion of oxidative metabolism, unlike exercise in normal air conditions.

**Figure 5:**
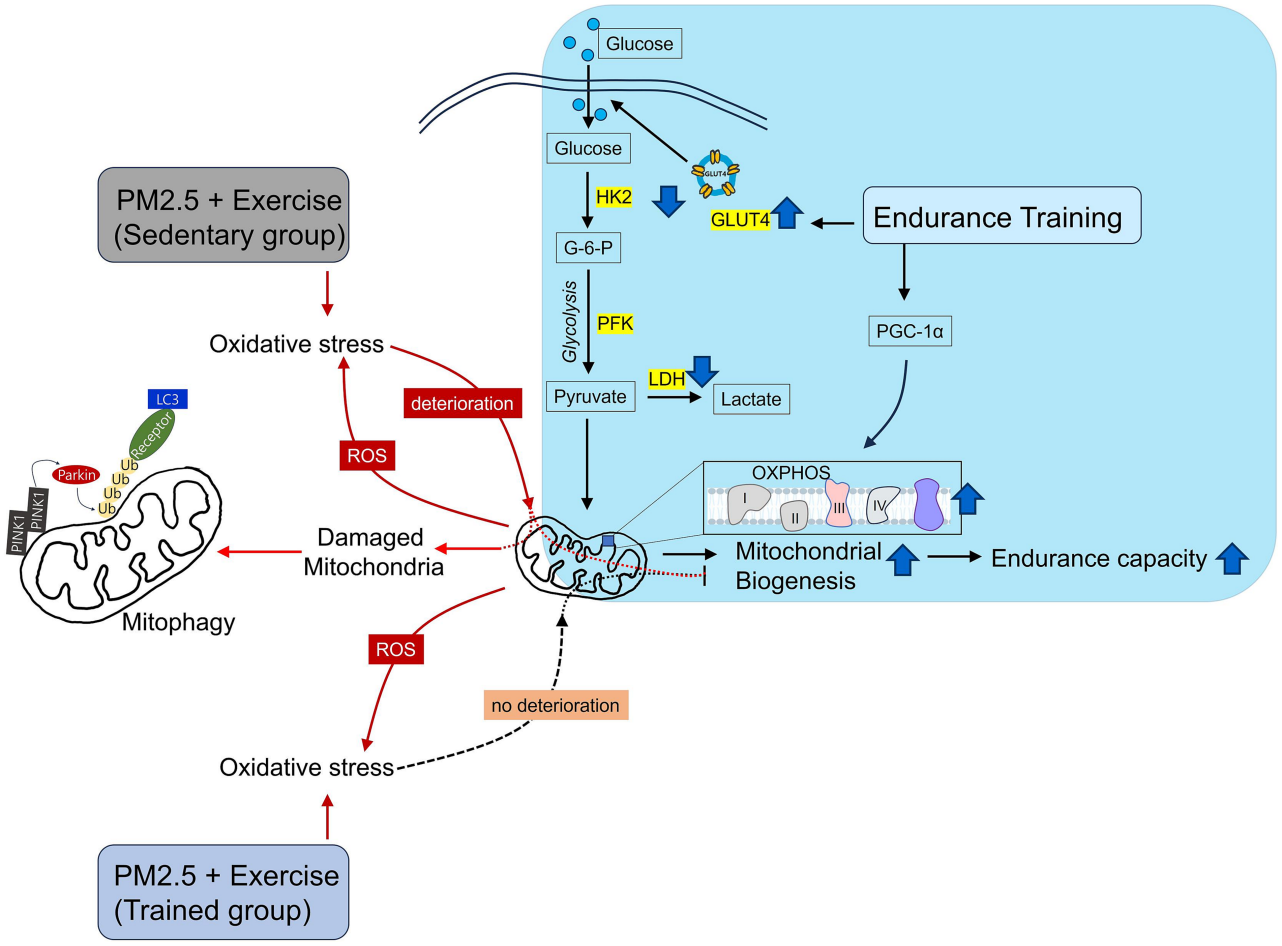
Schematic illustration of mitochondrial damage caused by exercise after ambient PM2.5 exposure. Endurance training improves skeletal muscle mitochondrial health. However, exercise after ambient PM2.5 exposure leads to mitochondrial deterioration. Nevertheless, endurance training prior to exercise after ambient PM2.5 exposure can prevent mitochondrial deterioration.

Mitochondria are dynamic organelles that undergo changes in shape and size (51). The process of regulating the dynamic structure of mitochondria is influenced by various intracellular signals and environmental changes, such as energy depletion, oxidative stress, mitochondrial damage, or cell survival (51, 52). Therefore, the dynamic structure of mitochondria plays a crucial role not only in regulating cellular energy supply and metabolism but also in influencing cell health and survival (52). Exercise increases proteins related to fusion (MFN1, MFN2, and OPA1) and fission (DRP1 and Fis1), leading to changes in mitochondrial morphology. These changes increase mitochondrial turnover, removing damaged mitochondria, and ensuring even energy distribution to all parts of the muscle (52). In this study, fusion and fission-related proteins, MFN1, and Drp1, increased (while MFN2, OPA1, and Fis1 remained unchanged), confirming that long-term endurance training can alter mitochondrial morphology (Figure 3A). However, these changes were suppressed by PM2.5 exposure and exercise, with similar trends observed for antioxidant capacity (Figure 4B) and PINK1-Parkin-mediated mitophagy (Figure 4C). PM2.5 refers to fine particulate matter with a diameter of 2.5 micrometers or less, containing chemical and toxic substances related to inflammation (9, 21). PM2.5 inhalation increases oxidative stress (12, 13), which reduces mitochondrial biogenesis and alters mitochondrial morphology, leading to mitochondrial dysfunction (53, 54). The findings of this study revealed that the enhanced mitochondrial function attributed to endurance training (ETR or EEX group) a decline within the EPE group (Figures 1–3). As shown in Figure 4A, this decrease is believed to stem from the heightened oxidative stress resulting from the brief exposure of the EPE group to PM2.5 inhalation. The responses to short-term PM2.5 inhalation and exercise, regardless of the presence of long-term endurance training, showed similar tendencies in the SPE and EPE groups. In terms of EEC, skeletal muscle metabolism, mitochondrial dynamics, and oxidative stress, the effects of exercise were either not evident or diminished. However, distinct responses were observed for mitophagy (Figure 4C) and mitochondrial damage (Figure 3B). The PINK1-Parkin pathway and Bax/Bcl2 ratio are critical cell signaling pathways related to mitophagy and apoptosis (55). The PINK1-Parkin pathway plays a crucial role in maintaining mitochondrial health by detecting mitochondrial damage and mediating apoptosis and mitophagy (55). Additionally, Bax and Bcl2, proteins located on the mitochondrial outer membrane, play essential roles in regulating apoptosis by, respectively, increasing and inhibiting the permeability of the mitochondrial outer membrane. The Bax/Bcl2 ratio changes in response to cellular stress in mitochondria, although this study found no significant changes in response to endurance training and PM2.5 exposure (Figure 4C). However, mitochondrial damage mediated by the PINK1-Parkin pathway significantly increased in the SPE group compared to other groups (Figures 3B, 4C). Particularly, the group treated with both PM2.5 exposure and exercise (EPE group), which exhibited similar responses to the SSED and EEX groups, showed no significant differences. These results mean that improvement of aerobic fitness by endurance training in normal air can prevent mitochondrial damage due to short-term exposure to fine dust. All these results are summarized in Figure 5.

It is important to note that the current study primarily focused on evaluating the effects of aerobic exercise, particularly on skeletal muscle mitochondria, after exposure to atmospherically relevant artificial PM2.5. However, it is crucial to recognize that when assessing the impacts of fine particulate matter exposure, the lungs emerge as one of the most significant organs. In cases of exposure to artificial PM2.5 (SPE and EPE), we observed an increase in lung inflammation and oxidative stress compared to conditions without exposure (SSED and EEX), as confirmed in Supplementary Figure 3. Interestingly, in contrast to skeletal muscle, the EPE group exhibited higher levels of inflammation and oxidative stress compared to the SPE group, suggesting the need for further research in this specific context.

## 5 Conclusion

In conclusion, increased oxidative stress by exposure to atmospherically relevant artificial PM2.5 may interfere with the effectiveness of short-term endurance exercise and cause mitochondrial damage. However, enhanced aerobic fitness and mitochondrial health by long-term endurance training may prevent mitochondrial damage from PM2.5 inhalation. Our findings suggest that regular exercise is required to promote overall health in a safe environment free from fine dust. The current study only examined the short-term effects of PM2.5 exposure and exercise. Physiological functions such as skeletal muscle metabolism and immune function may vary according to the duration of specific stresses (56). Therefore, verification of the long-term effects of PM2.5 exposure and/or exercise under various conditions is necessary.

## Data availability statement

The original contributions presented in the study are included in the article/Supplementary materials, further inquiries can be directed to the corresponding authors (sh5275@jbnu.ac.kr).

## Ethics statement

The animal study was approved by Institutional Animal Care and Use Committee of Jeonbuk National University (IACUC approval no. CBNU-2022-0067). The study was conducted in accordance with the local legislation and institutional requirements.

## Author contributions

WL: Data curation, Formal analysis, Methodology, Validation, Writing – original draft. ZW: Formal analysis, Validation, Data curation, Writing – review & editing. YG: Formal analysis, Writing – review & editing. H-SS: Formal analysis, Validation, Writing – review & editing. S-HK: Conceptualization, Investigation, Project administration, Writing – review & editing. YP: Conceptualization, Methodology, Project administration, Supervision, Writing – review & editing, Writing – original draft. SK: Conceptualization, Funding acquisition, Investigation, Methodology, Project administration, Supervision, Validation, Visualization, Writing – original draft, Writing – review & editing.
