# Supplementary material for: Effects of short-term exercise and endurance training on skeletal muscle mitochondria damage induced by particular matter, atmospherically relevant artificial PM2.5
Source: Front Public Health. 2024 Feb 28;12:1302175. doi: 10.3389/fpubh.2024.1302175 (PMC10933037; doi:10.3389/fpubh.2024.1302175)
Supplement: Supplementary file 1 [file Data_Sheet_1.pdf]

Supplementary Material

Supplementary Table 1. List of the chemical compositions, formula, and dry mass fractions of organic and inorganic species used in this study.

**Functional Group**	**Components**	**Formula**	**Density (g/cm^3) at 295 K***	**Dry mass fraction (%)**
Monocarboxylic acid	Acetate	$C_2H_3O_2$	1.05	6.25
Dicarboxylic acid	Oxalic acid	$C_2H_2O_4$	1.90	6.25
	Malonic acid	$C_3H_4O_4$	1.62	6.25
	Glutaric acid	$C_5H_8O_4$	1.35	6.25
Polyols	Glycerol	$C_3H_8O_3$	1.26	6.25
Sugars	Sucrose	$C_{12}H_{22}O_{11}$	1.59	6.25
Aromatics	2,5-Dihydroxybenzoic acid	$C_7H_6O_3$	1.55	6.25
Amino acid	Glycine	$C_2H_5O_2N$	1.61	6.25
Inorganic salts	Ammonium sulfate	$(NH_4)_2SO_4$	1.77	25
	Ammonium nitrate	NH_4NO_3	1.72	25

* Values of measured densities are from www.chemicalbook.com

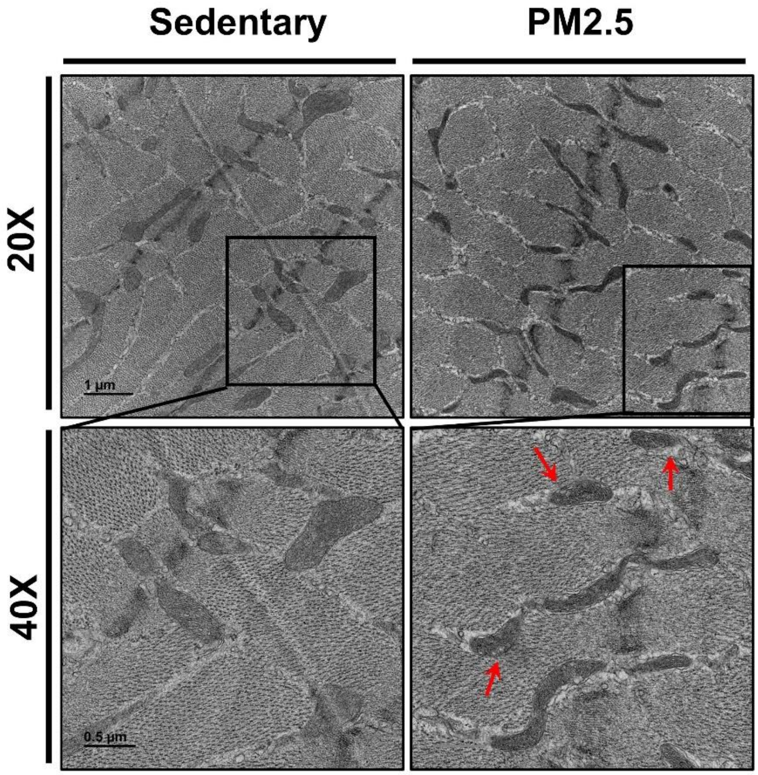

Supplementary Figure 1. TEM images for the confirmation of skeletal muscle mitochondrial damage in mice due to a total of three times exposures to ambient PM2.5, with 2 hours of exposure once a day on alternate days, at a concentration of 50.1 ± 8.1μg/m^3. Damaged mitochondria are indicated by red arrows.

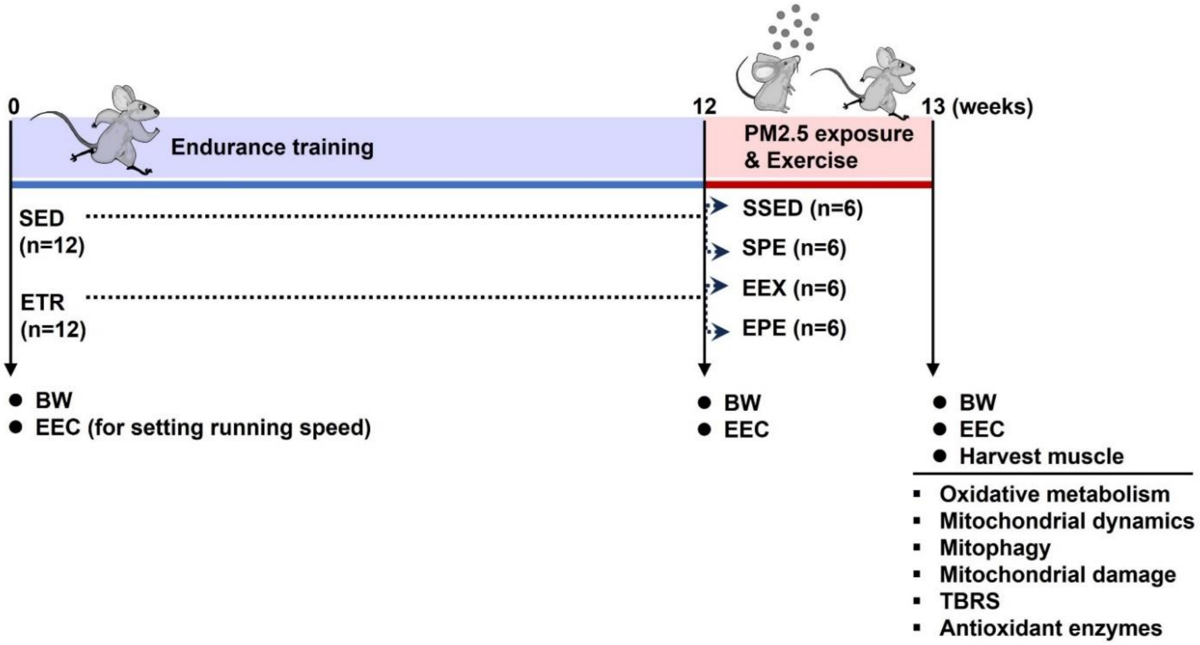

Supplementary Figure 2. Schematic overview of the experimental design for treatment and group classification according to study period and analysis items. SED, sedentary group; ETR, endurance training group; SSED, SED and sedentary group; SPE, SED and aerobic exercise after PM2.5 exposure; EEX, ETR and exercise; EPE, ETR and aerobic exercise after PM2.5; BW, Body weight; EEC, Endurance exercise capacity.

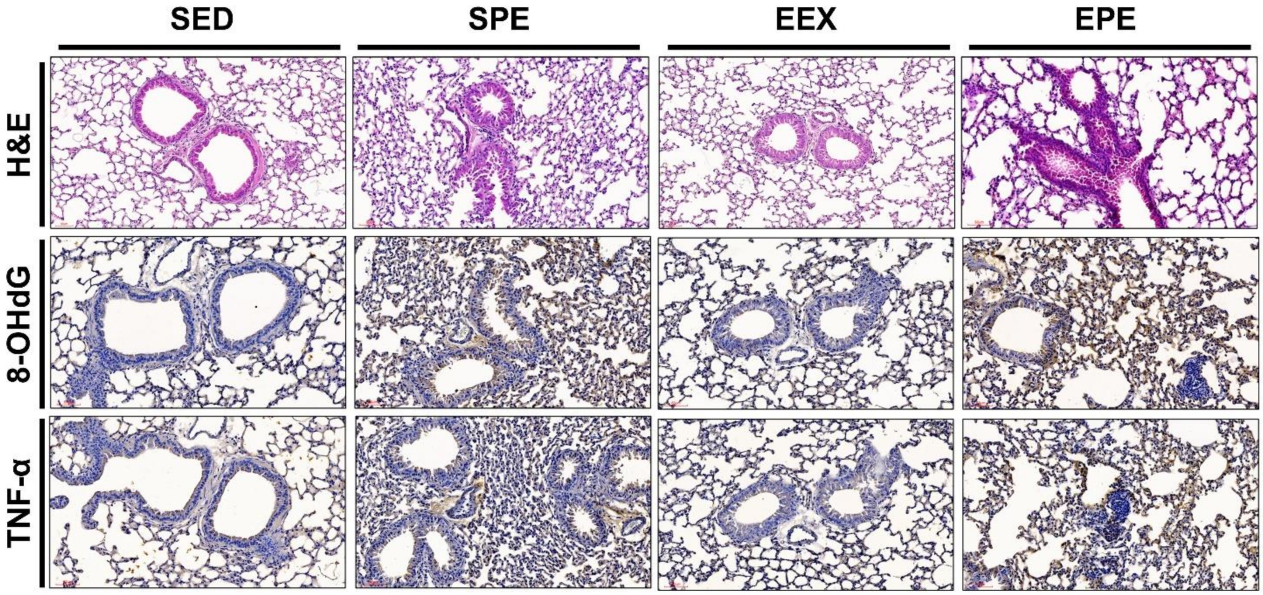

Supplementary Figure 3. Exposure to $PM_{2.5}$ impairs the lungs by inducing oxidative stress and inflammation. Immunohistochemical expression levels of 8-hydroxydeoxyguanosine (8-OHdG) and tumor necrosis factor α (TNF-α) along with H&E staining. Representative results are shown (n = 6). Scale bars: 60 µm.
